# Deep learning image reconstruction algorithm: impact on image quality in coronary computed tomography angiography

**DOI:** 10.1007/s11547-023-01607-8

**Published:** 2023-02-27

**Authors:** Domenico De Santis, Tiziano Polidori, Giuseppe Tremamunno, Carlotta Rucci, Giulia Piccinni, Marta Zerunian, Luca Pugliese, Antonella Del Gaudio, Gisella Guido, Luca Barbato, Andrea Laghi, Damiano Caruso

**Affiliations:** Radiology Unit, Department of Medical-Surgical Sciences and Translational Medicine, Sapienza University of Rome, Sant’Andrea University Hospital, Via di Grottarossa, 1035-1039, 00189 Rome, Italy

**Keywords:** Artificial intelligence, Deep learning, Image reconstruction, Coronary computed tomography angiography, CCTA

## Abstract

**Purpose:**

To perform a comprehensive intraindividual objective and subjective image quality evaluation of coronary CT angiography (CCTA) reconstructed with deep learning image reconstruction (DLIR) and to assess correlation with routinely applied hybrid iterative reconstruction algorithm (ASiR-V).

**Material and methods:**

Fifty-one patients (29 males) undergoing clinically indicated CCTA from April to December 2021 were prospectively enrolled. Fourteen datasets were reconstructed for each patient: three DLIR strength levels (DLIR_L, DLIR_M, and DLIR_H), ASiR-V from 10% to 100% in 10%-increment, and filtered back-projection (FBP). Signal-to-noise ratio (SNR) and contrast-to-noise ratio (CNR) determined objective image quality. Subjective image quality was assessed with a 4-point Likert scale. Concordance between reconstruction algorithms was assessed by Pearson correlation coefficient.

**Results:**

DLIR algorithm did not impact vascular attenuation (*P* ≥ 0.374). DLIR_H showed the lowest noise, comparable with ASiR-V 100% (*P* = 1) and significantly lower than other reconstructions (*P* ≤ 0.021).

DLIR_H achieved the highest objective quality, with SNR and CNR comparable to ASiR-V 100% (*P* = 0.139 and 0.075, respectively). DLIR_M obtained comparable objective image quality with ASiR-V 80% and 90% (*P* ≥ 0.281), while achieved the highest subjective image quality (4, IQR: 4–4; *P* ≤ 0.001). DLIR and ASiR-V datasets returned a very strong correlation in the assessment of CAD (*r* = 0.874, *P* = 0.001).

**Conclusion:**

DLIR_M significantly improves CCTA image quality and has very strong correlation with routinely applied ASiR-V 50% dataset in the diagnosis of CAD.

## Introduction

Coronary computed tomography angiography (CCTA) plays a pivotal role as non-invasive tool in the diagnosis of obstructive coronary artery disease (CAD), due to its widespread availability, high diagnostic accuracy, excellent negative predictive value, and continuous technical advancements [1–3]; additionally, thanks to a thorough assessment of coronary atherosclerosis burden, CCTA is able to predict future major adverse cardiac events [4]. Nevertheless, despite a trend in radiation dose reduction, radiation exposure still represents a major concern and there is ample room for improvements in patient safety [5–7].

Filtered back-projection (FBP) represented the standard reconstruction algorithm for over three decades, ensuring robust and time-effective CT images. However, this method does not maintain adequate image quality when applying dose reduction strategies. Such limitations have been overcome in 2009 by the implementation of iterative reconstruction (IR) algorithms. IR techniques, either hybrid (combined with FBP) or model-based (stand-alone), grant low-dose CT examinations with acceptable image noise. Nevertheless, along with their denoising capabilities, IR is burdened by modification of image texture, ultimately leading to over-smoothed images, and preventing their full exploitation [8–10].

Deep learning image reconstruction (DLIR) algorithms, based on deep convolutional neural networks, have been recently released by vendors holding promise for shorter reconstruction time and significantly reduced noise while preserving image texture [11–13]. DLIR applied to CCTA is currently under active investigation in different tasks, such as image optimization, classification, segmentation, prognosis and outcome prediction [14–16]; in particular, DLIR is achieving promising results compared to IR at specific strength levels [17–19]. Nevertheless, to the best of our knowledge, no previous investigations have assessed DLIR image quality in a broad comparison with IR and FBP.

Thus, the purpose of our study was to perform a comprehensive intraindividual objective and subjective image quality evaluation of CCTA reconstructed with DLIR and to assess correlation with routinely applied hybrid iterative reconstruction algorithm.

## Materials and methods

### Patient population

This prospective, single-center study was approved by local institutional review board and written informed consent was obtained from all patients. Consecutive patients who underwent clinically indicated CCTA for known or suspected CAD were enrolled from April to December 2021. Exclusion criteria were: (a) severe motion artifacts on CCTA, (b) contraindication to contrast medium injection, and (c) heart rate > 90 bpm.

Intravenous β-blocker (Metoprolol, 5 mg) was administrated to patients with heart rate > 75 bpm, after exclusion of contraindications. Nitrates (Trinitrine, 0.8 mg) were sublingual administrated to all patients in order to induce vasodilatation for a better evaluation of coronary arteries.

### Image acquisition

All CCTAs were performed in a cranio-caudal direction during end-inspiration, with retrospective ECG-gating, on a 128-slice CT scanner (GE Revolution EVO, GE Medical Systems, Milwaukee, WI). The following parameters were applied: detector collimation width of 0.625 mm, gantry rotation time of 0.6 s, spiral pitch automatically adjusted on heart rate and ranging from 0.16 to 0.30, and matrix of 512 × 512 pixels. Tube voltage and tube current modulation were fixed according to patient’s body mass index (BMI): 80 kV and 150 mA for patients with BMI < 30, 100 kV and 200 mA for patients with BMI > 30.

A fixed amount (50 mL) of non-ionic high-iodine concentration contrast medium (400 mgI/mL iomeprol, Iomeron 400; Bracco Imaging, Italy) was intravenously injected at a fixed flow rate of 5 mL/s through an 18-gauge antecubital access, by using an automated triple-syringe power injector (MEDRAD® Centargo CT Injection System; Bayer AG, Berlin, Germany), followed by saline chaser bolus of 40 mL at the same flow rate. Scan delay was determined using a bolus-tracking software program (SmartPrep, GE Healthcare): CCTA acquisition started after automatic minimum diagnostic delay as soon as the trigger attenuation threshold (100 HU) was reached into a region-of-interest (ROI) placed in the ascending aorta at the level of pulmonary arteries.

### Image reconstruction

Every examination was reconstructed at a thickness of 0.625 mm by means of three different algorithms: (1) FBP, (2) hybrid IR (ASiR-V, GE Healthcare) at strength levels from 10% to 100% with 10%-increments, and (3) DLIR (TrueFidelity™, GE Healthcare) at three strength levels: low, medium, and high (DLIR_L, DLIR_M, and DLIR_H, respectively). Thus, fourteen different image datasets have been eventually generated for each CT examination. TrueFidelity™ applies a deep neural network, previously trained with high-quality FBP datasets, able to discern image noise from signal and to reconstruct CT images by selectively suppressing noise [13].

### Objective image quality analysis

Quantitative measurements were performed on all fourteen reconstructed image datasets, by a radiologist with 5 years of experience in cardiovascular imaging, on a dedicated workstation (Advantage Workstation 4.7, GE Healthcare) for each patient and in all reconstructed datasets.

In axial sections, ROIs were drawn in the left pectoral muscle, ascending aorta (at the origin of the left main coronary artery), left main artery, left anterior descending artery, circumflex artery, and right coronary artery, carefully avoiding inclusion of the vessel wall and atherosclerotic plaques. Image noise was defined as the standard deviation (SD) of the ROI drawn in the pectoral muscle.

All ROIs were placed three times, and measurements have been averaged to minimize measurement inaccuracies. Consistency on ROIs placement throughout the datasets was ensured by applying the copy and paste function of the workstation.

Signal-to-noise ratio (SNR) was calculated as follows:

$${\text{SNR}} = \frac{{{\text{HU}}_{{{\text{artery}}}} }}{{{\text{SD}}_{{{\text{muscle}}}} }}$$

Contrast-to-noise ratio (CNR) was calculated as follows:

$${\text{CNR}} = \frac{{{\text{HU}}_{{{\text{artery}}}} - {\text{HU}}_{{{\text{muscle}}}} }}{{{\text{SD}}_{{{\text{muscle}}}} }}$$

### Subjective image quality analysis

Two radiologists with 8 and 12 years of experience in CCTA, blinded to reconstruction protocol, performed subjective image analysis on ASiR-V 50%, ASiR-V 100%, DLIR_M, and DLIR_H datasets, in consensus reading. Datasets were selected for subjective image quality based on routine clinical practice (ASiR-V 50%), results of objective image quality analysis (ASiR-V 100% and DLIR_H), and vendor recommendations (DLIR_M). Images were evaluated with standard window setting (width, 1200 HU; level, 240 HU) but freely adjustable to suit readers’ preferences. Ambient lighting condition was kept constant at approximately 35–40 lx.

To minimize recall bias, images were evaluated in a randomized order and no more than two different reconstructed datasets from each patient were analyzed during each interpretation, maintaining a time interval of 7 days between sessions.

Image quality focused on plaques was assessed using an ordinal 4-point Likert scale from 1 to 4 (1, poor; 2, adequate; 3, good; and 4, excellent contour delineation). Coronary segments were considered diagnostic when image quality was deemed adequate, good, or excellent (scores 2–4) [20], in case of multiple plaques per segment, the plaque with the highest degree of stenosis was used for further analysis.

### Correlation between DLIR and ASiR-V

The analysis was performed on ASiR-V 50% (dataset routinely used in clinical practice) and DLIR_M (dataset achieving the highest overall subjective image quality) by the two radiologists who had performed the subjective image quality analysis, in consensus reading, after a 2-week interval. The coronary artery tree was analyzed based on the segmentation described by the SCCT guidelines for the interpretation and reporting of CCTA [21]. Axial images and curved multiplanar reformats were used for image evaluation, and the window level setting was freely modifiable. Each coronary segment was visually scored for CAD as follows: absent (0% luminal narrowing), non-obstructive CAD (1–49% luminal narrowing), and obstructive CAD (≥ 50% luminal narrowing) [22]; in case of multiple lesions per segment, the coronary segment was classified based on the lesion with the highest degree of stenosis.

### Statistical analysis

Statistical analyses were performed using commercially available software (IBM Corp. Released 2017. IBM SPSS Statistics for Macintosh, Version 25.0. Armonk, NY: IBM Corp). The Kolmogorov–Smirnov test was used to assess the normality of data distribution.

Continuous variables were expressed as mean ± SD if normally distributed and as median and interquartile range (IQR) if non-normally distributed; categorical variables were expressed as median and IQR.

Vascular attenuation values, image noise, and image quality of the different reconstruction datasets were compared using repeated-measures ANOVA test or Friedman test, as appropriate.

Correlation between DLIR_M and ASiR-V 50% datasets was measured by means of Pearson correlation coefficients. A *P* value < 0.05 was considered to indicate a statistically significant result; post hoc pairwise comparisons were adjusted for multiple comparisons by the Bonferroni correction.

## Results

### Patient population

**Fig. 1:**
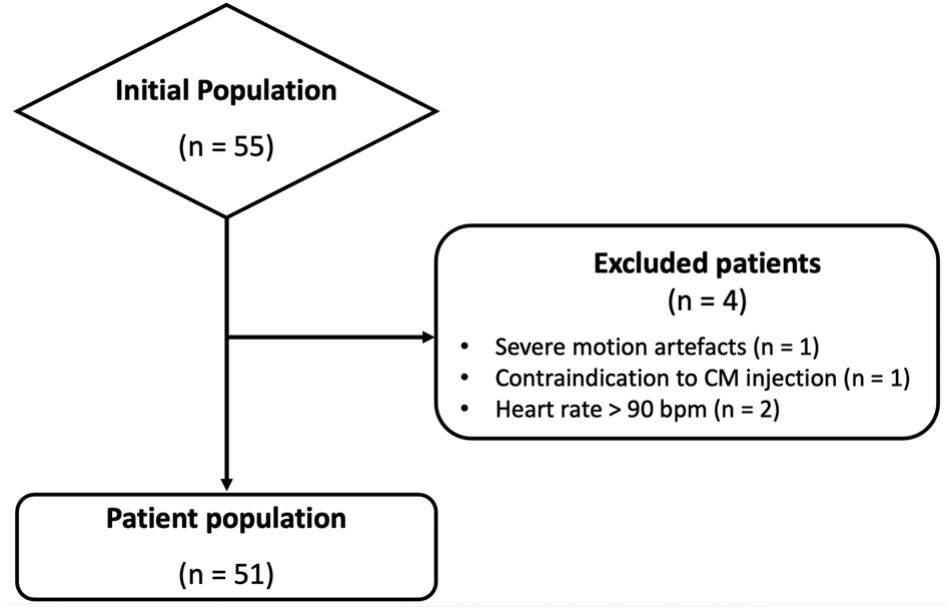
Flow diagram of patient population. [CM, contrast medium]

Comprehensive results of patient characteristics are reported in Table 1, and corresponding flow diagram is depicted in Fig. 1.

**Table 1:** Patient characteristics

Parameter	Value
No. of patients	51
Age (years)*	64 ± 15 (18–84)
Male-to-female ratio	29:22
Body mass index (kg/m^2^)*	26.6 ± 4.9 (16.3–37.2)
*Cardiovascular risk factors*^*†*^	
Hypertension	37 (72.5)
Dyslipidemia	18 (35.3)
Current of former smoking	14 (27.5)
Diabetes mellitus	10 (19.6)
Familiar history of CAD	31 (60.8)
*Cardiac history n (%)*	
Previous MI	6 (11.8)
Stable angina	4 (7.8)
Revascularization	5 (9.8)
*Medications*	
Statin	14 (27.5)
β-blocker	17 (33.3)
Calcium antagonist	6 (11.8)
Diuretic	1 (2)
Insulin	1 (2)
Oral hypoglycemic agents	3 (5.9)
Others	17 (33.3)

*CAD* Coronary artery disease, *MI* Myocardial infarction

*Data are mean ± standard deviation (range)

^†^Data are number of patients (%)

Of 55 patients initially identified, 1 was excluded due severe motion artifacts, 1 due to contraindication to CM injection, and 2 were excluded due to heart rate > 90 bpm. Hence, the final population eligible for image quality analysis consisted of 51 patients (22 females), with a mean age of 64 ± 15 years (range 18–84 years) and a mean BMI of 26.6 ± 4.9 kg/m^2^ (range 16.3–37.2). The mean heart rate measured during CCTA acquisitions was 63 ± 9 bpm.

### Objective image quality

Comprehensive objective image quality scores are summarized in Table 2.

**Table 2:** Objective image quality scores of FBP, ASiR-V, and DLIR reconstructions

	FBP	ASiR-V 10%	ASiR-V 20%	ASiR-V 30%	ASiR-V 40%	ASiR-V 50%	ASiR-V 60%
Attenuation*	626.5 ± 156.1	609.7 ± 138.2	606.1 ± 137.5	602.9 ± 137.8	600.5 ± 137.8	596.7 ± 138.4	597.1 ± 137.7
Noise^†^	41.7 (17.9–72.6)	27.6 (12.6–45.2)	24.0 (12.1–41.1)	21.0 (10.4–36.5)	18.5 (9.7–32.5)	16.1 (8.6–29.2)	14.7 (7.8–27.0)
SNR^†^	13.7 (8.4–37.6)	22.9 (13.9–56.4)	24.1 (15.3–68.1)	27.3 (17.1–68.9)	29.1 (18.8–75.0)	32.2 (19.9–83.3)	36.4 (22.6–91.1)
CNR^†^	12.2 (7.4–32.8)	20.4 (12.5–52.8)	21.4 (13.7–57.6)	24.6 (13.3–64.4)	26.5 (16.9–69.6)	29.3 (18.8–77.2)	33.0 (20.5–83.2)

	ASiR-V 70%	ASiR-V 80%	ASiR-V 90%	ASiR-V 100%	DLIR_L	DLIR_M	DLIR_H
Attenuation*	594.8 ± 140.3	596.0 ± 139.0	594.9 ± 139.0	594.4 ± 139.5	613.2 ± 136.1	612.1 ± 136.4	611.3 ± 137.9
Noise^†^	13.7 (7.0–24.7)	12.7 (6.3–22.5)	11.2 (5.6–20.1)	10.4 (5.0–17.9)	15.8 (6.5–24.7)	13.9 (5.6–21.6)	9.7 (4.5–15.5)
SNR^†^	39.6 (24.7 100.5)	44.4 (27.7–113.1)	49.7 (30.2–129.9)	55.7 (32.6–144.0)	41.5 (23.5–103.1)	49.5 (28.3–121.4)	65.1 (37.7–159.4)
CNR^†^	35.4 (22.3–91.9)	39.0 (24.3–104.4)	44.6 (27.3–117.0)	51.0 (30.0–134.6)	36.7 (21.3–101.4)	43.7 (25.7–114.0)	59.1 (34.0–153.4)

^†^Data are median (interquartile range)

*ASiR-V* Hybrid iterative reconstruction algorithm, *CNR* Contrast-to-noise ratio, *DLIR* Deep learning image reconstruction algorithm, *FBP* Filtered back-projection, *SNR* Signal-to-noise ratio

*Data are mean ± standard deviation

**Fig. 2:**
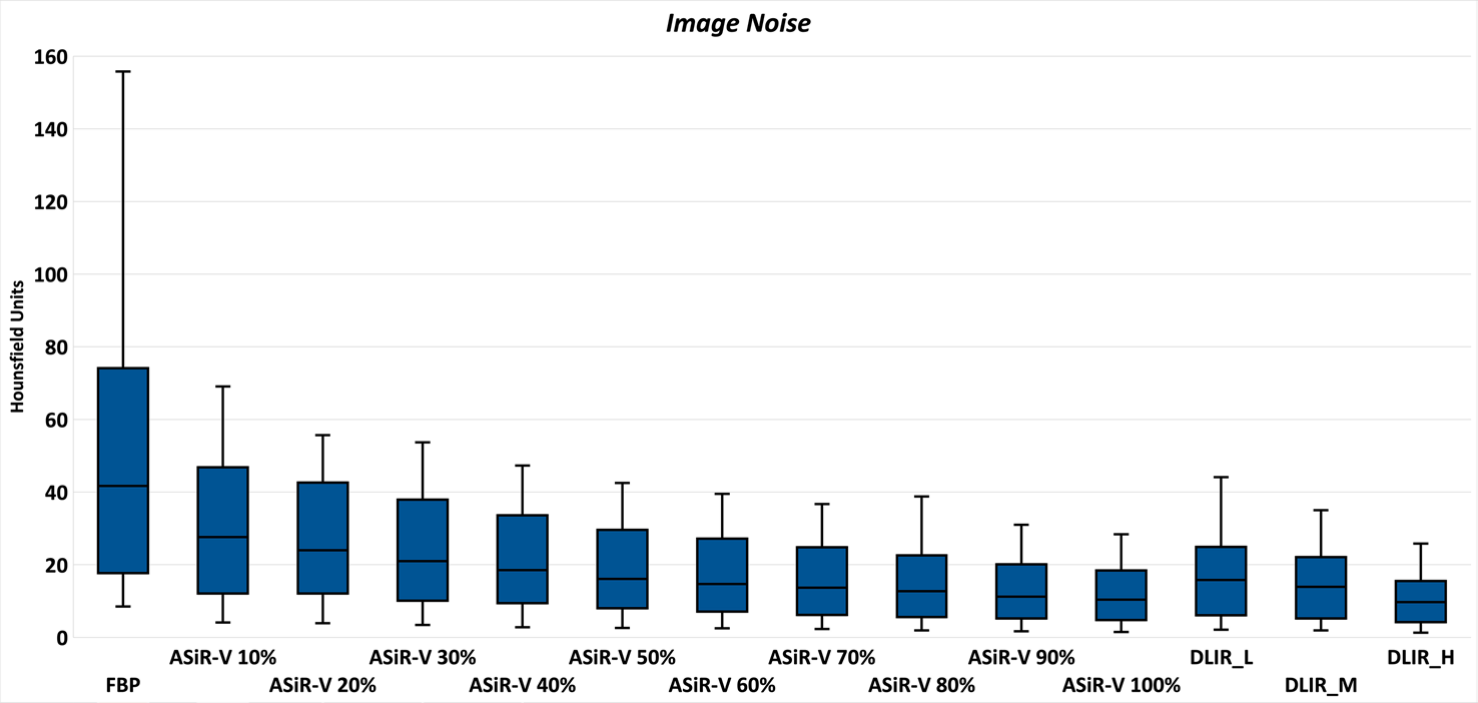
Box-and-whisker plots for image noise. DLIR_H achieved the lowest image noise, followed by ASiR-V 100%, DLIR_M, DLIR_L, and all the remaining ASiR-V datasets. Boxes represent the middle 50% of the data and solid lines represent the median, whiskers represent minimum and maximum values

**Fig. 3:**
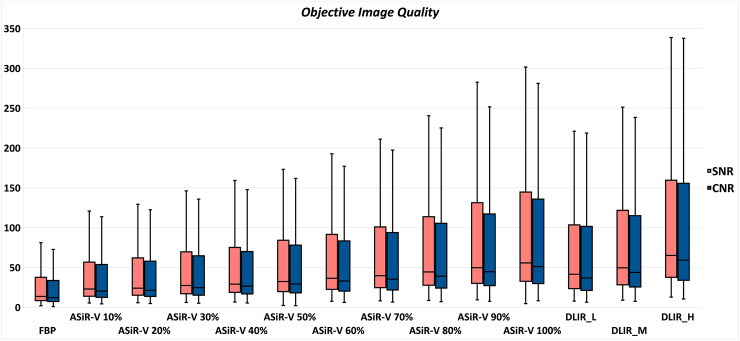
Box-and-whisker plots for SNR and CNR. DLIR_H reached the highest SNR and CNR, comparable with ASiR-V 100% and significantly higher than DLIR_M, DLIR_L, and all the remaining ASiR-V datasets. DLIR_M showed comparable values with ASiR-V 80% and ASiR-V 90%, and higher values than DLIR_L. DLIR_L dataset showed comparable values with ASiR-V 60% and ASiR-V 70%. Boxes represent the middle 50% of the data and solid lines represent the median, whiskers represent minimum and maximum values. [CNR: contrast-to-noise ratio; SNR: signal-to-noise ratio]

A total of 714 datasets were analyzed. DLIR algorithm did not affect vascular attenuation values compared with FBP and every ASiR-V reconstruction (*P* ≥ 0.374). Graphical representation of image noise and objective image quality is shown in Figs. 2 and 3, respectively.

The lowest noise was obtained by DLIR_H (median: 9.7, IQR: 4.5–15.5), comparable with ASiR-V 100% (median: 10.4; IQR: 5.0–17.9; *P* = 1) and significantly lower than DLIR_M (median: 13.9; IQR: 5.6–21.6; *P* = 0.011), DLIR_L (median: 15.8; IQR: 6.5–24.7; *P* < 0.001), and all the remaining ASiR-V datasets (*P* ≤ 0.021). DLIR_M dataset showed comparable image noise with ASiR-V 80% and ASiR-V 90% (*P* = 1), and lower noise than DLIR_L (*P* = 0.023). DLIR_L dataset exhibited comparable image noise with ASiR-V 60% and ASiR-V 70% (*P* = 1). The highest image noise was measured with FBP dataset (median: 41.7; IQR: 17.9–72.6), significantly different than every DLIR reconstructions (*P* < 0.001).

The highest SNR was achieved by DLIR_H (median: 65.1, IQR: 37.7–159.4), comparable with ASiR-V 100% (median: 55.7; IQR: 32.6–144.0; *P* = 0.139) and significantly higher than DLIR_M (median: 49.5; IQR: 28.3–121.4; *P* < 0.001), DLIR_L (median: 41.5; IQR: 23.5–103.1; *P* < 0.001), and all the remaining ASiR-V datasets (*P* < 0.001). DLIR_M dataset showed comparable SNR with ASiR-V 80% and ASiR-V 90% (*P* = 1), and higher SNR than DLIR_L (*P* < 0.001). DLIR_L dataset showed comparable SNR with ASiR-V 60% (*P* = 0.157) and ASiR-V 70% (*P* = 1). The lowest SNR was measured with FBP reconstruction (median: 13.7; IQR: 8.4–37.6), significantly different than every DLIR reconstructions (*P* < 0.001).

The highest CNR was achieved by DLIR_H (median: 59.1, IQR: 34.0–153.4), comparable with ASiR-V 100% (median: 51.0; IQR: 30.0–134.6; *P* = 0.075) and significantly higher than DLIR_M (median: 43.7; IQR: 25.7–114.0; *P* < 0.001), DLIR_L (median: 36.7; IQR: 21.3–101.4; *P* < 0.001), and all the remaining ASiR-V datasets (*P* < 0.001). DLIR_M dataset showed comparable CNR with ASiR-V 80% (*P* = 0.281) and ASiR-V 90% (*P* = 1), and higher CNR than DLIR_L (*P* < 0.001). DLIR_L dataset showed comparable CNR with ASiR-V 60% (*P* = 0.113) and ASiR-V 70% (*P* = 1). The lowest CNR was measured with FBP reconstruction (median: 12.2; IQR: 7.4–32.8), significantly different than every DLIR reconstructions (*P* < 0.001). Pairwise comparisons between groups are reported in Table 3 and in Table 4, for CNR and SNR, respectively.

**Table 3:** Pairwise comparisons of CNR achieved by FBP, ASiR-V, and DLIR reconstructions

	FBP	ASiR-V 10%	ASiR-V 20%	ASiR-V 30%	ASiR-V 40%	ASiR-V 50%	ASiR-V 60%	ASiR-V 70%	ASiR-V 80%	ASiR-V 90%	ASiR-V 100%	DLIR_L	DLIR_M	DLIR_H
FBP		.118*	< . 001	< . 001	< . 001	< . 001	< . 001	< . 001	< . 001	< . 001	< . 001	< . 001	< . 001	< . 001
ASiR-V 10%	.118*		1*	< . 001	< . 001	< . 001	< . 001	< . 001	< . 001	< . 001	< . 001	< . 001	< . 001	< . 001
ASiR-V 20%	< . 001	1*		1*	< . 001	< . 001	< . 001	< . 001	< . 001	< . 001	< . 001	< . 001	< . 001	< . 001
ASiR-V 30%	< . 001	< . 001	1*		.422*	< . 001	< . 001	< . 001	< . 001	< . 001	< . 001	< . 001	< . 001	< . 001
ASiR-V 40%	< . 001	< . 001	< . 001	.422*		1*	< . 001	< . 001	< . 001	< . 001	< . 001	< . 001	< . 001	< . 001
ASiR-V 50%	< . 001	< . 001	< . 001	< . 001	1*		.155*	< . 001	< . 001	< . 001	< . 001	< . 001	< . 001	< . 001
ASiR-V 60%	< . 001	< . 001	< . 001	< . 001	< . 001	.155*		.054*	< . 001	< . 001	< . 001	.113*	< . 001	< . 001
ASiR-V 70%	< . 001	< . 001	< . 001	< . 001	< . 001	< . 001	.054*		.051*	< . 001	< . 001	1*	< . 001	< . 001
ASiR-V 80%	< . 001	< . 001	< . 001	< . 001	< . 001	< . 001	< . 001	.051*		.006	< . 001	.023	.281*	< . 001
ASiR-V 90%	< . 001	< . 001	< . 001	< . 001	< . 001	< . 001	< . 001	< . 001	.006		.022	< . 001	1*	< . 001
ASiR-V 100%	< . 001	< . 001	< . 001	< . 001	< . 001	< . 001	< . 001	< . 001	< . 001	.022		< . 001	< . 001	.075*
DLIR_L	< . 001	< . 001	< . 001	< . 001	< . 001	< . 001	.113*	1*	.023	< . 001	< . 001		< . 001	< . 001
DLIR_M	< . 001	< . 001	< . 001	< . 001	< . 001	< . 001	< . 001	< . 001	.281*	1*	< . 001	< . 001		< . 001
DLIR_H	< . 001	< . 001	< . 001	< . 001	< . 001	< . 001	< . 001	< . 001	< . 001	< . 001	.075*	< . 001	< . 001	

*Non-statistically significant *P* values

*ASiR-V* Hybrid iterative reconstruction algorithm, *DLIR* Deep learning image reconstruction algorithm, *FBP* Filtered back-projection

**Table 4:** Pairwise comparisons of SNR achieved by FBP, ASiR-V, and DLIR reconstruction

	FBP	ASiR-V 10%	ASiR-V 20%	ASiR-V 30%	ASiR-V 40%	ASiR-V 50%	ASiR-V 60%	ASiR-V 70%	ASiR-V 80%	ASiR-V 90%	ASiR-V 100%	DLIR_L	DLIR_M	DLIR_H
FBP		.139*	< . 001	< . 001	< . 001	< . 001	< . 001	< . 001	< . 001	< . 001	< . 001	< . 001	< . 001	< . 001
ASiR-V 10%	.139*		1*	< . 001	< . 001	< . 001	< . 001	< . 001	< . 001	< . 001	< . 001	< . 001	< . 001	< . 001
ASiR-V 20%	< . 001	1*		1*	< . 001	< . 001	< . 001	< . 001	< . 001	< . 001	< . 001	< . 001	< . 001	< . 001
ASiR-V 30%	< . 001	< . 001	1*		.357*	< . 001	< . 001	< . 001	< . 001	< . 001	< . 001	< . 001	< . 001	< . 001
ASiR-V 40%	< . 001	< . 001	< . 001	.357*		.992*	< . 001	< . 001	< . 001	< . 001	< . 001	< . 001	< . 001	< . 001
ASiR-V 50%	< . 001	< . 001	< . 001	< . 001	.992*		.241*	< . 001	< . 001	< . 001	< . 001	< . 001	< . 001	< . 001
ASiR-V 60%	< . 001	< . 001	< . 001	< . 001	< . 001	.241*		.023	< . 001	< . 001	< . 001	.157*	< . 001	< . 001
ASiR-V 70%	< . 001	< . 001	< . 001	< . 001	< . 001	< . 001	.023		.009	< . 001	< . 001	1*	< . 001	< . 001
ASiR-V 80%	< . 001	< . 001	< . 001	< . 001	< . 001	< . 001	< . 001	.009		.317*	< . 001	.001	1*	< . 001
ASiR-V 90%	< . 001	< . 001	< . 001	< . 001	< . 001	< . 001	< . 001	< . 001	.317*		.004	< . 001	1*	< . 001
ASiR-V 100%	< . 001	< . 001	< . 001	< . 001	< . 001	< . 001	< . 001	< . 001	< . 001	.004		< . 001	< . 001	.139*
DLIR_L	< . 001	< . 001	< . 001	< . 001	< . 001	< . 001	.157*	1*	.001	< . 001	< . 001		< . 001	< . 001
DLIR_M	< . 001	< . 001	< . 001	< . 001	< . 001	< . 001	< . 001	< . 001	1*	1*	< . 001	< . 001		< . 001
DLIR_H	< . 001	< . 001	< . 001	< . 001	< . 001	< . 001	< . 001	< . 001	< . 001	< . 001	.139*	< . 001	< . 001	

*Non-statistically significant *P* values

*ASiR-V* Hybrid iterative reconstruction algorithm, *DLIR* Deep learning image reconstruction algorithm, *FBP* Filtered back-projection

### Subjective image quality

**Fig. 4:**
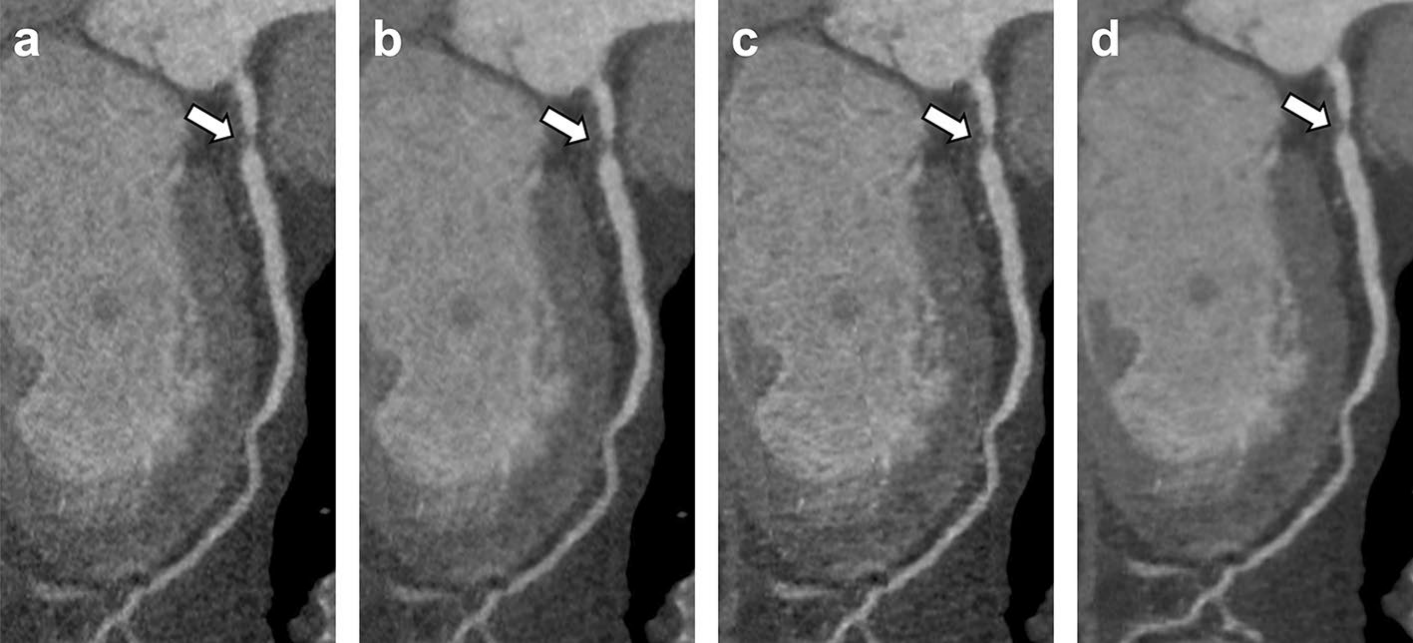
A 64-year-old male with familiar history of CAD. Curved multiplanar reformations reconstructed with ASiR-V 50% (**a**), ASiR-V 100% (**b**), DLIR_M (**c**), and DLIR_H (**d**) show obstructive (> 50%) soft plaque of proximal LAD; DLIR_M achieved the highest subjective image quality score. [ASiR-V: hybrid iterative reconstruction algorithm; CAD: coronary artery disease; DLIR: deep learning image reconstruction algorithm; LAD: left anterior descending artery; window width, 1200 HU; level, 240 HU]

Subjective image quality scores and corresponding pairwise comparisons are showed in Table 5. No examination reconstructed with the DLIR datasets was deemed of non-diagnostic image quality. DLIR_M returned the highest overall median image quality (score: 4; IQR: 4–4), significantly higher than all the other reconstructions (*P* ≤ 0.001). DLIR_H and ASiR-V 50% datasets achieved comparable results (scores: 3, *P* = 0.085); followed by ASiR-V 100% (score: 2; IQR: 2–3); Fig. 4.

**Table 5:** Subjective image quality scores of ASiR-V and DLIR reconstructions, with related pairwise comparisons

	Score^†^	Pairwise comparisons			
		ASiR-V 50%	ASiR-V 100%	DLIR_M	DLIR_H
ASiR-V 50%	3 (3–3)		.004	.001	.085*
ASiR-V 100%	2 (2–3)	.004		.001	< .001
DLIR_M	4 (4–4)	.001	.001		< .001
DLIR_H	3 (3–4)	.085*	< .001	< .001	

*Non-statistically significant *P* values

*ASiR-V* Hybrid iterative reconstruction algorithm, *DLIR* Deep learning image reconstruction algorithm

^†^Data are median (interquartile range)

### Correlation between DLIR and ASiR-V

**Fig. 5:**
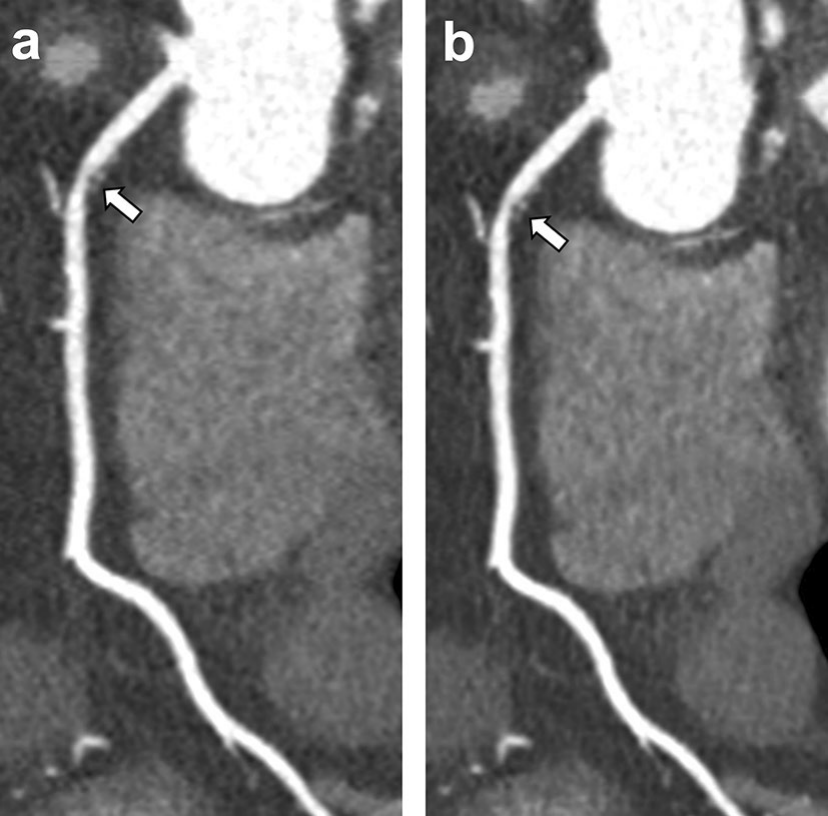
A 81-year-old female with family history of CAD. Curved multiplanar reformation of the ASiR-V 50% dataset (**a**) and DLIR_M dataset (**b**) show a concentric mixed plaque determining non-obstructive (1–49%) stenosis of the proximal RCA (arrow). The two datasets show an overall very strong correlation in the assessment of CAD. [CAD: coronary artery disease; DLIR_M: medium-strength deep learning image reconstruction algorithm; RCA: right coronary artery; window width, 1200 HU; level, 240 HU]

A total of 721 coronary segments were assessed for the presence of coronary stenosis. With routinely applied ASiR-V 50% dataset, no stenoses were reported in 584 segments, stenoses 1–49% were found in 101 segments, while stenoses ≥ 50% were found in 36 segments. With DLIR_M, no stenoses were reported in 577 segments, stenoses 1–49% were found in 107 segments, while stenoses ≥ 50% were found in 37 segments, with a concordance between the two datasets of 98%, 73%, and 76%, respectively, and an overall very strong correlation (*r* = 0.874 *P* = 0.001) Fig. 5.

## Discussion

The aim of our study was to perform a comprehensive intraindividual image quality evaluation of CCTA reconstructed with DLIR and to assess correlation between ASiR-V and DLIR in the diagnosis of CAD. Our investigation demonstrated that DLIR did not affect vascular attenuation compared with ASiR-V and FBP, DLIR_H showed the lowest noise, comparable with ASiR-V 100% and significantly lower than every other reconstruction. DLIR_H achieved the highest objective image quality, with SNR and CNR comparable with ASiR-V 100%. DLIR_M returned the highest subjective image quality, significantly higher than all the other reconstructions. Additionally, a very strong correlation was found between DLIR_M and ASiR-V 50% datasets in the diagnosis of CAD.

An adequate tradeoff between diagnostic image quality and radiation dose has always been a crucial aim of CT technical advancements. A radiation output reduction of the X-ray tube not counterweighted by compensating strategies translates into non-diagnostic images, due to unacceptable increase in image noise. Lack of computational power prevented IR technology to be clinically feasible until 2009, when they started replacing FBP as the reconstruction modality of choice [23]. Despite providing significant radiation dose reduction and overall increase in image quality, IR algorithms tend to alter image texture, generating over-smoothed “plastic-like” images. Imaging over-smoothing increases as function of strength of IR [24]; therefore, middle strength levels (usually from 50% to 70%) are commonly implemented in clinical practice, representing a good tradeoff between image noise and texture. The constant increase in computation power, along with increasing availability of big data, paved the way for DLIR algorithms. Two major CT vendors, GE Healthcare and Canon Medical System, had their DLIR algorithms cleared by the FDA, both based on a deep neural network, respectively, trained with high-quality FBP images [13] and model-based IR datasets [12]. Both algorithms are under extensive investigation and are achieving promising results: Recent clinical studies have documented DLIR capability of generating images with lower image noise and superior image quality compared to IR; favorable results have been obtained in CCTA [17, 25–27], abdominal CT [28–32], chest CT examinations [33–35], and brain CT scans [36, 37].

DLIR is a more stable reconstruction method compared to IR since its performances are less influenced by variation of dose and levels of reconstructions, proven effective in reducing image noise and improving image quality without alteration of the typical FBP noise texture [38]. DLIR_H has been proved effective in achieving 37% noise reduction compared to DLIR_L and 40% noise reduction compared to ASiR-V 50% in a phantom experiment, our investigation translated these results in vivo with solid consistency, demonstrating a denoising power up to 39% compared to DLIR_L and 40% compared to ASiR-V 50%. Our widespread comparison of fourteen different datasets shed further light on DLIR performance in reducing image noise, demonstrating DLIR_H better performances compared to FBP (77% noise reduction) and ASiR-V from 10% to 90% (65% to 13% noise reduction). Specifically, such findings are also in accordance with recent studies focused on CCTA and reporting a DLIR_H denoising performance of 54% compared with ASiR-V 60% [26] and 43% compared with ASiR-V 70% [17].

As results of DLIR denoising efficacy, our investigation demonstrated that SNR and CNR increased as a function of DLIR strength, peaking at DLIR_H: median image quality measured with DLIR_H was roughly 44% higher than ASiR-V 60% and 39–40% higher than ASiR-V 70%, in accordance with existing literature [17, 26]. DLIR_H reached also greater objective image quality than stronger ASiR-V levels; however, despite achieving 14% higher score than ASiR-V 100%, the two values were comparable.

Our study also demonstrated that DLIR at medium strength levels provided the highest subjective image quality. No examinations reconstructed with DLIR datasets were deemed of unacceptable image quality; however, the median image quality of DLIR_M was deemed excellent, followed by DLIR_H and ASiR-V 50%, with no statistical differences between these two datasets. These results differ from Benz and colleagues [17], who reported DLIR_H to be superior or comparable to DLIR_M. Nevertheless, in our investigation readers pointed out minor blurring of small plaque contours with DLIR_H, ultimately leading to loss of image details. These subtle effects might explain the highest performances of DLIR_M, also considering that similar blurring has been reported for DLIR_M algorithm in abdominal setting [29]. The alteration of noise texture and consequent imaging over-smoothing is a well-known limitation of IR algorithms, representing the main reason for opting to lower strengths level in clinical practice. As opposite with ASiR-V, the investigated DLIR is trained with FBP data, has little to no impact noise texture and reconstructs crisper datasets characterized by diagnostic equal accuracy.

The high image quality ensured by DLIR, along with its a time-effective reconstruction process (≤ 50 s for axial CCTAs [38]), paves the way for its implementation in routine clinical practice. Dedicated CCTA acquisition protocols can be designed to exploit DLIR capabilities of reconstructing high-quality of low-dose examinations [17, 18, 27]. The improved image quality ensured by DLIR might also allow the use of dedicated low-volume contrast media injection protocols, particularly useful in elderly individuals or in patients with heart failure and impaired renal function. Hence, the application of DLIR algorithm might be beneficial in clinical practice to allow gentler radiation dose protocols without detrimental effect on image quality and diagnostic accuracy.

The findings of this study should be seen in light of some limitations. First, the investigated DLIR algorithm and the obtained results are vendor-specific; therefore, our findings might not be directly transposable to other DLIR algorithms. However, all the investigation performed so far has achieved promising preliminary results. Second, despite we included patient who had undergone coronary stenting, we did not perform a subgroup analysis to specifically test the DLIR performances on stented segments. Further diagnostic accuracy was not evaluated, because invasive coronary catheterization was not routinely available as a reference standard; therefore, our results need to be strengthened with larger patients cohorts and multi-institutional investigations.

In conclusion, DLIR algorithm at medium strength level significantly improves CCTA image quality and has very strong correlation with routinely applied ASiR-V 50% dataset in the diagnosis of CAD.
